# Long non‐coding RNA FTH1P3 activates paclitaxel resistance in breast cancer through miR‐206/ABCB1

**DOI:** 10.1111/jcmm.13679

**Published:** 2018-07-03

**Authors:** Ruoming Wang, Tengteng Zhang, Zhen Yang, Chunxia Jiang, Jingjing Seng

**Affiliations:** ^1^ Department of Thyroid and Breast Surgery The First People's Hospital of Shangqiu Shangqiu China; ^2^ Department of Oncology The First People's Hospital of Shangqiu Shangqiu China; ^3^ Department of Gastrointestinal Surgery The First Affiliated Hospital of Zhengzhou University Zhengzhou China; ^4^ Editorial Board of Journal of Zhengzhou University Zhengzhou China; ^5^ Department of Breast Surgery The First Affiliated Hospital of Zhengzhou University Zhengzhou China

**Keywords:** ABCB1, breast cancer, FTH1P3, miR‐206, paclitaxel resistance

## Abstract

Emerging evidence has indicated the important function of long non‐coding RNAs (lncRNAs) in tumour chemotherapy resistance. However, the underlying mechanism is still ambiguous. In this study, we investigate the physiopathologic role of lncRNA ferritin heavy chain 1 pseudogene 3 (FTH1P3) on the paclitaxel (PTX) resistance in breast cancer. Results showed that lncRNA FTH1P3 was up‐regulated in paclitaxel‐resistant breast cancer tissue and cells (MCF‐7/PTX and MDA‐MB‐231/PTX cells) compared with paclitaxel‐sensitive tissue and parental cell lines (MCF‐7, MDA‐MB‐231). Gain‐ and loss‐of‐function experiments revealed that FTH1P3 silencing decreased the 50% inhibitory concentration (IC50) value of paclitaxel and induced cell cycle arrest at G2/M phase, while FTH1P3‐enhanced expression exerted the opposite effects. In vivo, xenograft mice assay showed that FTH1P3 silencing suppressed the tumour growth of paclitaxel‐resistant breast cancer cells and ABCB1 protein expression. Bioinformatics tools and luciferase reporter assay validated that FTH1P3 promoted ABCB1 protein expression through targeting miR‐206, acting as a miRNA “sponge.” In summary, our results reveal the potential regulatory mechanism of FTH1P3 on breast cancer paclitaxel resistance through miR‐206/ABCB1, providing a novel insight for the breast cancer chemoresistance.

## 1 INTRODUCTION

Gradually, breast cancer has been one of the most common gynaecological malignant tumours and the leading cause of cancer‐related death of females worldwide.^1^, ^2^ In spite of the endeavour and progress on clinical therapeutic strategies, the long‐term clinical prognosis and mortality rate are still unsatisfactory.^3^ Among these influence factors, chemoresistance is a major obstacle and often causes the poor treatment or failure.^4^ Increasing evidence has demonstrated that breast cancer cells have developed chemoresistance against multiple first‐line chemotherapy drugs, including paclitaxel, cisplatin, docetaxel, gemcitabine and so on.^5^ The chemoresistance of multiple‐drugs has become the major impediment for the clinical effects and application for these drugs.

Paclitaxel is an effective first‐line chemotherapy drug in clinical oncology, which is specific microtubule‐stabilizing agent.^6^ Paclitaxel is isolated from Taxus brevifolia and acts as one of the most effective plant‐derived anticancer drugs.^7^ The molecular mechanism for paclitaxel chemotherapy is to cause cell cycle arrest at G2/M phase and induce the cell apoptosis.^8^ However, the treatment effect of paclitaxel is going worse due to the chemoresistance caused by multifarious factors. Therefore, the most strategy of treatment is to clarify the underlying mechanisms of chemoresistance.

Long non‐coding RNAs (lncRNAs) are type transcripts with more than 200 nucleotides without non‐protein coding capacity.^9^ In series of tumour tissue and cells, lncRNAs have been verified to be dysregulated and contribute to the multifaceted molecular regulation.^10^, ^11^ Moreover, lncRNAs have been validated to participate in the formation of tumour chemotherapy resistance through regulating critical molecular pathway and protein transcription.^12^ For example, in breast cancer cells, lncRNA‐ROR down‐regulation inhibits the epithelial mesenchymal transition and promotes the sensibility towards tamoxifen by regulating miR‐205/ZEB1/ZEB2 expression.^13^

LncRNA FTH1P3 is a novel identified ncRNA in cancer. For example, FTH1P3 was up‐regulated in uveal melanoma cell lines and tissues, and the elevated expression of FTH1P3 promoted uveal melanoma cell proliferation, cell cycle and migration through suppressing miR‐224‐5p.^14^ Here, we demonstrate that lncRNA ferritin heavy chain 1 pseudogene 3 (FTH1P3) expression is enriched in paclitaxel‐resistant breast cancer tissue samples and cells. FTH1P3 acted as a competing endogenous RNA (ceRNA) to sponge miR‐206 and increase ABCB1 protein expression. Taken together, our results illuminate the FTH1P3/miR‐206/ABCB1 pathway in the chemoresistance of breast cancer.

## 2 MATERIALS AND METHODS

### 2.1 Clinical tissue samples

From March 2015 to August 2016, 30 patients with breast cancer (15 paclitaxel‐sensitive patients and 15 paclitaxel‐resistant patients), who received surgical treatment in the First Affiliated Hospital of Zhengzhou University and the First People's Hospital of Shangqiu, were recruited in this research. The tumorous tissue was rapidly frozen at −80°C for using. This study was approved by the Research Ethics Committee of First Affiliated Hospital of Zhengzhou University and the First People's Hospital of Shangqiu. The informed written consents were collected from all patients.

### 2.2 Paclitaxel‐resistant breast cancer cells construction

Human breast cancer cell lines (MCF‐7, MDA‐MB‐231, MDA‐MB‐468, MDA‐MB‐453) and normal human breast epithelial cell (MCF‐10A) were purchased from ATCC (Manassas, VA, USA). The human breast cancer cell lines were cultured in RPMI 1640 medium supplemented with 10% foetal bovine serum and cultured at 37°C. The paclitaxel (PTX)‐resistant breast cancer cell lines (MCF‐7/PTX, MDA‐MB‐231/PTX) were constructed by continuous increasing coculture of paclitaxel. Then, the paclitaxel resistance was maintained with 1 μmol/L paclitaxel culture. Cellular assays were performed when cells in exponentially growing phase.

### 2.3 Paclitaxel sensitivity assay

Paclitaxel sensitivity was determined using CCK‐8 assay by measuring the IC50 value (paclitaxel concentration causing 50% decrease in absorbance compared with the control). Briefly, cells (MCF‐7/PTX, MDA‐MB‐231/PTX) were seeded in 96‐well plates at a density of 1 × 10^4^ cells per well for 24‐hour incubation after transfection. After incubation with paclitaxel treatment, the cell viability was measured using cell Counting Kit‐8 (Dojindo, Japan) according to the manufacturer's instructions.

### 2.4 Quantitative real‐time polymerase chain reaction (qRT‐PCR)

Total RNA was extracted from cells and tissue according to specification (Promega, Madison, WI., USA). The concentration and purity of RNA were measured at 260/280 nm using ultraviolet spectrophotometer by the standard of OD260/OD280 ratio of 1.6‐2.0. cDNA was reversely transcribed from RNA using SuperScript First‐Stand Synthesis system (Invitrogen, Carlsbad, Calif, US). qRT‐PCR was performed using ABI7500 quantitative PCR instrument. Primer sequences were synthesized by Sangon Biotech (Shanghai) as following: FTH1P3, forward, 5′‐TGACTACAGTCTTACCCCATCCT‐3′, reverse 5′‐CTGATAGCCACCTGAAATGCG‐3′; miR‐206, forward, 5′‐ACAACAAGGACCGGTTGCAGA‐3′, reverse, 5′‐GGGCATACATCGGCTAATACA‐3′; GAPDH, forward, 5′‐TCCACCACCCTGTTGCTGTA‐3′, reverse 5′‐ACCACAGTCCATGCCATCAC‐3′. Finally, the RNA expression levels were quantified with SYBR (Applied Biosystems, USA) on 7500 Real‐Time PCR System (Applied Biosystems, USA). The relative expression was normalized to the expression of GAPDH using 2^−ΔΔ*C*t^ method. The experiment was performed in triplicate.

### 2.5 Plasmid construction and transfection

Full‐length complementary DNA of FTH1P3 was synthesized by GenScript Biomedical (Shanghai, China) and cloned into the pcDNA3.1 (+) vector (Invitrogen) according to the manufacturer's instructions. A total of 5 × 10^5^ cells were seeded into each well of a 6‐well plate and transfected with shRNAs upon reaching 80%‐90% confluence. Recombinant plasmid pcDNA‐FTH1P3 and negative control plasmid (pcDNA‐NC) were constituted and, respectively, transfected into MCF‐7/PTX and MDA‐MB‐231/PTX cells using Lipofectamine 2000 (Invitrogen) according to the instruction manual. Transfection of si‐FTH1P3/si‐NC and miR‐206 inhibitor was performed using Lipofectamine 2000.

### 2.6 Flow cytometry

Cell cycle analysis was performed using cell cycle analysis kit (Lianke, Shanghai, China). Briefly, cells were digested with trypsin and washed with phosphate‐buffered saline (PBS). Cells (2 × 10^5^) cells were suspended with 50 μL binding buffer and then treated with cell cycle agents for 48 hours. Then, the adherent cells were washed and trypsinized, and centrifuged at 12 000 g for 5 minutes. After centrifugation, DNA staining was performed with 10 mg PI/mL PBS and 2.5 mg Ag DNase‐free RNase/mL PBS for 30 minutes. Cell cycle profiles were generated with Modifit software (BD Biosciences).

### 2.7 Western blot

The total protein was lysed by RIPA buffer (Sigma‐Aldrich) added with protease inhibitors cocktail (Roche). After that, the isolated protein was transferred to sodium dodecyl sulphate‐polyacrylamide gel electrophoresis (SDS‐PAGE), and then to PVDF membrane (Millipore, Billerica, MA, USA). The membranes were washed with TBST and blocked with 5% non‐fat milk powder and incubated with primary antibody, anti‐ABCB1 (1:1000 dilution, Abcam), at room temperature for 2 hour and overnight at 4°C. The membrane was incubated with second antibody (horseradish peroxidase‐conjugated goat anti‐rabbit) at room temperature for 2 hours. Finally, these blots were detected using an EZ‐ECL chemiluminescence detection kit for HRP (Biological Industries, Beit‐Haemek, Israel).

### 2.8 Luciferase reporter assay

HEK‐293T cells (1 × 104) were seeded into each well of 48‐well plate and cotransfected with luciferase reporter (10 ng) and miR‐206 mimics (50 nmol/L) or negative control using Lipofectamine 2000 (Invitrogen). 48 hours after transfection, luciferase activity assay was measured using the Dual‐Luciferase Reporter Assay System (Promega, Madison, WI, USA) and GloMax 20/20 LUMINOMETER (promega). Renilla luciferase activity was normalized against firefly luciferase activities. Results represented the triplicate independent experiments.

### 2.9 Xenograft mice assay in vivo

Ten male BALB/c nude mice (4 weeks old) were maintained under pathogen free conditions. The xenograft in vivo assay was approved by the Institutional Animal Care and Use Committee of Affiliated Cancer Hospital of Zhengzhou University and the First People's Hospital of Shangqiu. MCF‐7/PTX cells (5x10^5^ cells/100ul) were transfected with lentivirus‐mediated sh‐FTH1P3 or sh‐vector and injected subcutaneously into the posterior flank of nude mice. Tumour volume was measured using caliper following the formula length × width^2^/2. The tumour weight was weighed after the mice were killed.

### 2.10 Statistical analysis

All data were generated from tripartite independent experiments and presented as the mean ± SD. Statistical analyses were performed using SPSS 18.0 and GraphPad software. Differences between groups were analysed using Student's *t* test or chi‐square test analysis. Statistical significance was set as *P* < .05.

## 3 RESULTS

### 3.1 LncRNA FTH1P3 was up‐regulated in paclitaxel‐resistant breast cancer tissue and cells

**Figure 1:**
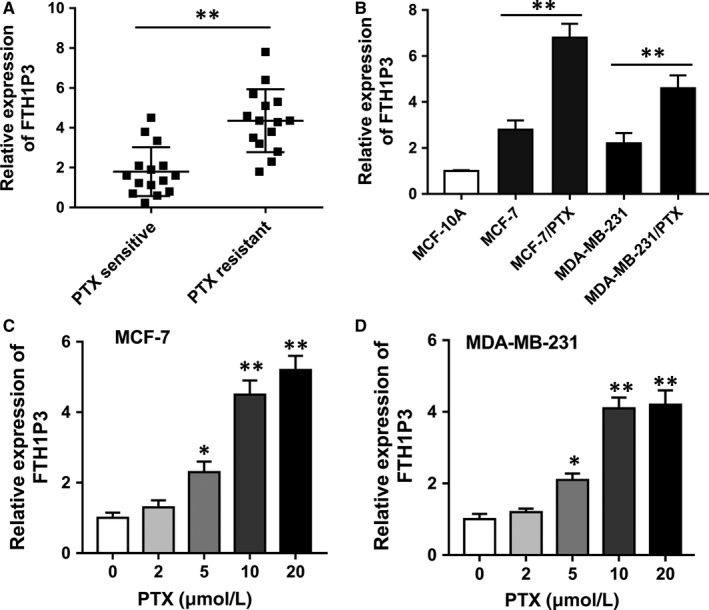
LncRNA FTH1P3 was up‐regulated in paclitaxel‐resistant breast cancer tissue and cells. A, RT‐PCR showed the lncRNA FTH1P3 expression level in 30 cases of breast cancer samples, including 15 cases of paclitaxel‐sensitive samples and 15 cases of paclitaxel‐resistant samples. B, RT‐PCR showed the lncRNA FTH1P3 expression levels in paclitaxel‐resistant breast cancer cell lines (MCF‐7/PTX, MDA‐MB‐231/PTX) and parental cell lines (MCF‐7, MDA‐MB‐231). C, FTH1P3 expression in MCF‐7 cells treated with different doses of paclitaxel. D, FTH1P3 expression in MDA‐MB‐231 cells treated with different doses of paclitaxel. Data were expressed as mean ± SD. *P < .05, **P < .01 represent statistically difference

To verify the expression level of lncRNA FTH1P3 in paclitaxel‐resistant breast cancer tissue, RT‐PCR was performed in 30 cases of breast cancer samples (15 cases of paclitaxel‐sensitive samples and 15 cases of paclitaxel‐resistant samples). Results showed that lncRNA FTH1P3 was up‐regulated in paclitaxel‐resistant breast cancer tissue compared with that in paclitaxel‐sensitive samples (Figure 1A). Then, lncRNA FTH1P3 was increased in paclitaxel‐resistant breast cancer cell lines (MCF‐7/PTX, MDA‐MB‐231/PTX) compared to their parental cell lines (MCF‐7, MDA‐MB‐231) (Figure 1B). Moreover, RT‐PCR showed that FTH1P3 expression levels were increased in MCF‐7 and MDA‐MB‐231 cells treated with different doses of paclitaxel, presenting the dose‐depended manner of paclitaxel resistance and FTH1P3 expression (Figure 1C and D). Taken together, results revealed that lncRNA FTH1P3 was up‐regulated in paclitaxel‐resistant breast cancer tissue and cells, suggesting the underlying molecular role in breast cancer chemoresistance.

### 3.2 LncRNA FTH1P3 activated the paclitaxel sensitivity of breast cancer cells and induced the G2/M phase arrest

**Figure 2:**
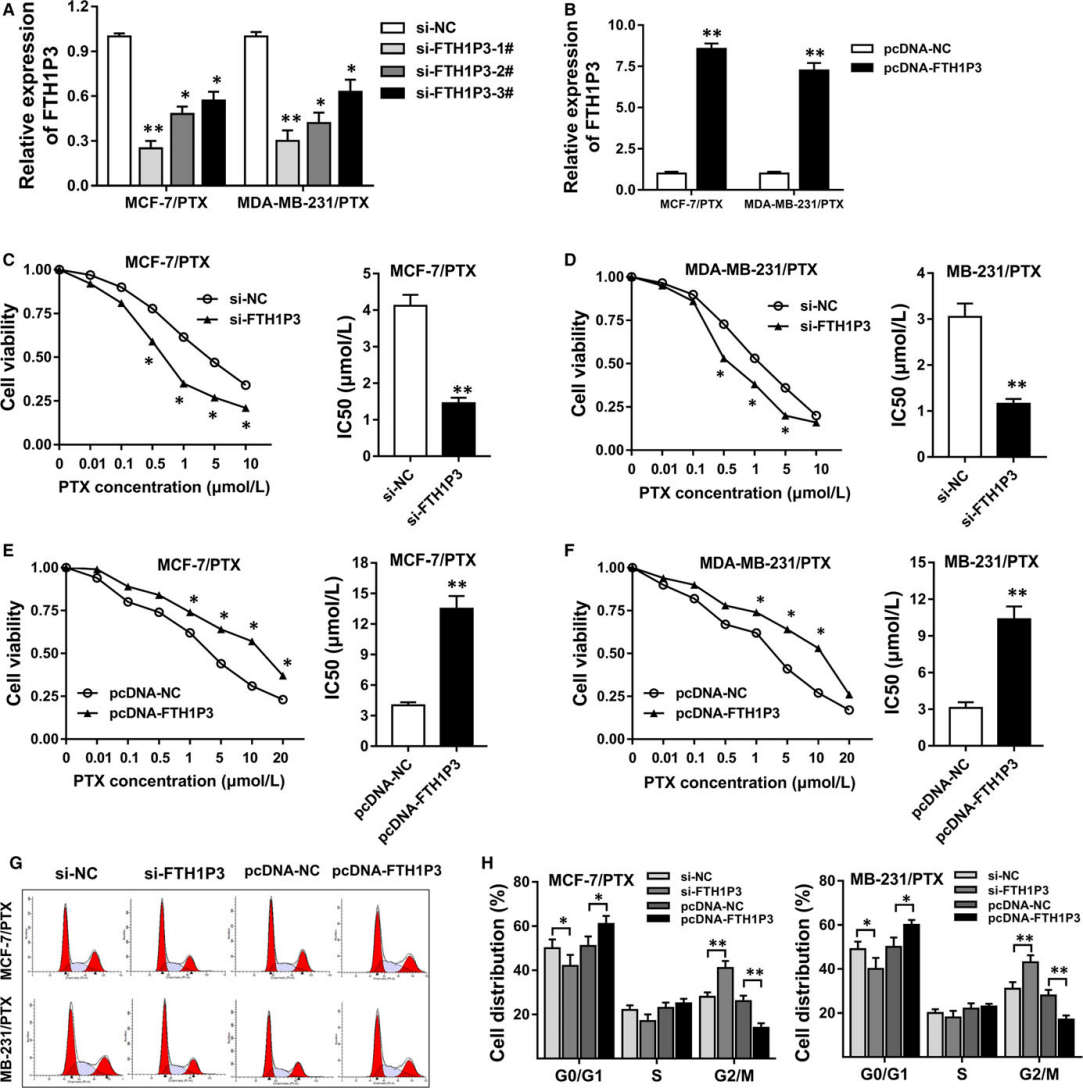
LncRNA FTH1P3 activated the paclitaxel sensitivity of breast cancer cells and induced the G2/M phase arrest. A, Synthesized small interfering RNA (siRNA) targeting FTH1P3 was transfected into MCF‐7/PTX and MDA‐MB‐231/PTX cells. FTH1P3 expression was measured using RT‐PCR. B, RT‐PCR showed the FTH1P3 expression in MCF‐7/PTX and MDA‐MB‐231/PTX cells transfected with plasmids containing FTH1P3 sequence. C, The 50% inhibitory concentration (IC50) value of paclitaxel in MCF‐7/PTX cells transfected with si‐FTH1P3 (1.87 μmol/L) or si‐NC (4.21 μmol/L). D, The IC50 value of paclitaxel in MDA‐MB‐231/PTX cells transfected with si‐FTH1P3 (1.23 μmol/L) or si‐NC (3.07 μmol/L). E, The IC50 value of paclitaxel in MCF‐7/PTX cells transfected with pcDNA‐FTH1P3 (12.83 μmol/L) or pcDNA‐NC (4.39 μmol/L). F, The IC50 value of paclitaxel in MDA‐MB‐231/PTX cells transfected with pcDNA‐FTH1P3 (9.86 μmol/L) or pcDNA‐NC (3.23 μmol/L). G, Images of flow cytometry assay. H, The cell distribution in MCF‐7/PTX and MDA‐MB‐231/PTX cells. Data were expressed as mean ± SD. *P < .05, **P < .01 represent statistically difference

Previous results had indicated that lncRNA FTH1P3 was up‐regulated in paclitaxel‐resistant breast cancer tissue and cells. In further experiments, we synthesized and transfected small interfering RNA (siRNA) targeting FTH1P3 and plasmids to down‐regulate (Figure 2A) and up‐regulate (Figure 2B) FTH1P3 expression levels in MCF‐7/PTX and MDA‐MB‐231/PTX cells. The 50% inhibitory concentration (IC50) value was measured using CCK‐8 assay for the response of FTH1P3 up‐regulation and down‐regulation towards paclitaxel. Results showed that the IC50 value of paclitaxel in MCF‐7/PTX (Figure 2C, 4.21 μmol/L vs 1.87 μmol/L) and MDA‐MB‐231/PTX (Figure 2D, 3.07 μmol/L vs 1.23 μmol/L) cells was down‐regulated in FTH1P3 silencing transfection compared to control group. On the other hand, the IC50 value of paclitaxel was up‐regulated in FTH1P3‐overexpressed transfected MCF‐7/PTX (Figure 2E, 4.39 μmol/L vs 12.83 μmol/L) and MDA‐MB‐231/PTX (Figure 2F, 3.23 μmol/L vs 9.86 μmol/L) compared to control group. Flow cytometry assay showed that the cell distribution was significantly increased at G2/M phase in FTH1P3 silencing transfection compared to control group (Figure 2G and H). Besides, the cell distribution was significantly decreased at G2/M phase in FTH1P3‐overexpressed transfection compared to control group, indicating the role of FTH1P3 on G2/M phase arrest. Overall, all the data concluded that lncRNA FTH1P3 activated the paclitaxel sensitivity and induced the G2/M phase arrest of breast cancer cells in vitro.

### 3.3 LncRNA FTH1P3 silencing suppressed the tumour growth of paclitaxel‐resistant breast cancer cells and ABCB1 protein in vivo

**Figure 3:**
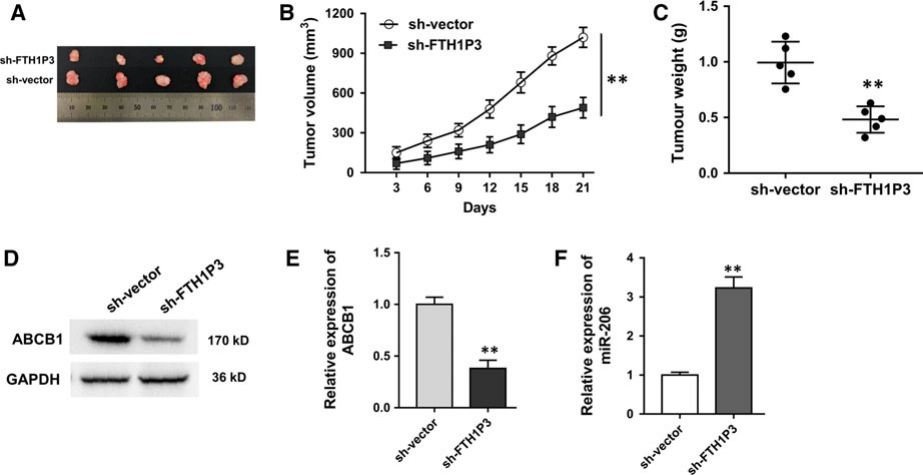
LncRNA FTH1P3 silencing suppressed the tumour growth of paclitaxel‐resistant breast cancer cells and ABCB1 protein in vivo. A, Images of tumour neoplasm in xenograft mice samples injected with MCF‐7/PTX transfected with sh‐FTH1P3 or empty vector. B, Tumour volume of neoplasm measured every 3 days after subcutaneous injection. C, Tumour weight of neoplasm excised from mice after they were killed. D, Images of Western blot band of ABCB1 protein. E, Quantitative value of ABCB1 protein expression. F, RT‐PCR showed the expression of miR‐206 in FTH1P3 knockdown group compared to empty vector group. Data were expressed as mean ± SD. *P < .05, **P < .01 represent statistically difference

It had been verified that lncRNA FTH1P3 was up‐regulated in paclitaxel‐resistant breast cancer cells and FTH1P3 regulated the paclitaxel sensitivity of breast cancer cells in vitro. Then, xenograft mice assay in vivo was performed using MCF‐7/PTX cells to test the role of FTH1P3 on tumour growth (Figure 3A). Results showed that FTH1P3 knockdown significantly decreased the tumour volume compared to empty vector group (Figure 3B). Besides, FTH1P3 knockdown significantly down‐regulated tumour weight compared to empty vector group (Figure 3C). In the tumour tissue sample, Western blot showed that ABCB1 protein was decreased in FTH1P3 knockdown group compared to empty vector group (Figure 3D and E). RT‐PCR showed that miR‐206 expression was increased in FTH1P3 knockdown group compared to empty vector group (Figure 3F). In summary, results indicated that FTH1P3 silencing suppressed the tumour growth of paclitaxel‐resistant breast cancer cells and ABCB1 protein in vivo.

### 3.4 Bioinformatics tools revealed that miR‐206 targeted 3′‐UTR of FTH1P3

**Figure 4:**
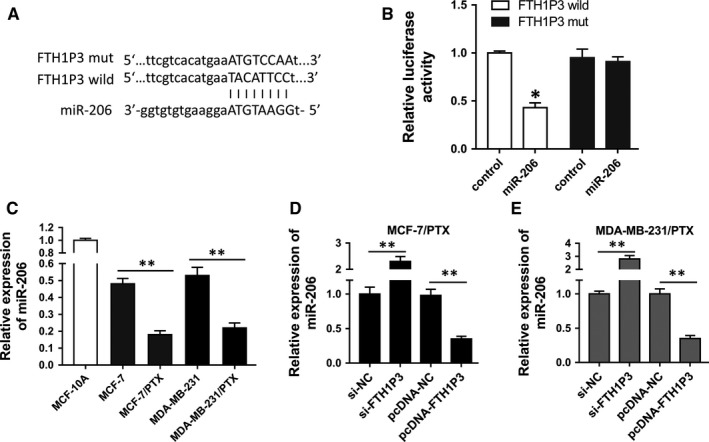
miR‐206 target 3′‐UTR of FTH1P3. A, Schematic diagrams of miR‐206, FTH1P3 wild‐type and FTH1P3 mutant type. B, Luciferase reporter assay showed the luciferase vitality in the combination within miR‐206 mimics/control and FTH1P3 wild‐type/mutant type. C, RT‐PCR showed the miR‐206 expression MCF‐7/PTX and MDA‐MB‐231/PTX cells compared with their parental cells. D, E, RT‐PCR showed the miR‐206 expression in MCF‐7/PTX and MDA‐MB‐231/PTX cells transfected with si‐FTH1P3 or pcDNA‐FTH1P3. Data were expressed as mean ± SD. *P < .05, **P < .01 represent statistically difference

Previous experiments revealed that lncRNA FTH1P3 and ABCB1 protein were closely correlated. To explore the potential relationship within FTH1P3 and ABCB1 protein, bioinformatics tools were performed. Results showed that miR‐206 shared complementary binding sites with 3′‐untranslated regions (3′‐UTR) of FTH1P3 (Figure 4A). Luciferase reporter assay confirmed the molecular binding within FTH1P3 and miR‐206 (Figure 4B). In breast cancer cells, PCR showed that miR‐206 expression was decreased in paclitaxel‐resistant breast cancer cells (MCF‐7/PTX, MDA‐MB‐231/PTX) compared with their parental cells (Figure 4C). Moreover, miR‐206 expression was increased in paclitaxel‐resistant cells transfected with si‐FTH1P3, while it was decreased in cells transfected with pcDNA‐FTH1P3 (Figure 4D and E). Therefore, results verified that miR‐206 targeted 3′‐UTR of FTH1P3.

### 3.5 FTH1P3 positively regulated ABCB1 expression via miR‐206

**Figure 5:**
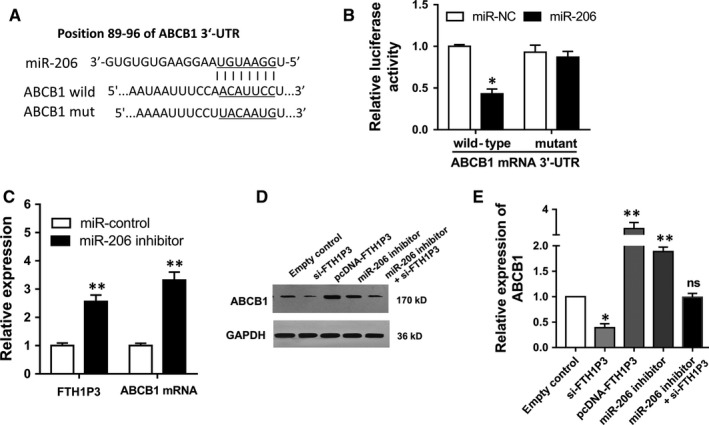
FTH1P3 positively regulated ABCB1 expression via miR‐206. A, Schematic diagrams of miR‐206, ABCB1 wild‐type and ABCB1 mutant type. B, Luciferase reporter assay showed the luciferase vitality in the combination within miR‐206 mimics/control and ABCB1 wild‐type/mutant type. C, RT‐PCR showed the ABCB1 and FTH1P3 expression in MCF‐7/PTX cells transfected with miR‐206 inhibitor. D, E, Western blots showed the ABCB1 protein expression in MCF‐7/PTX cells transfected with si‐FTH1P3, pcDNA‐FTH1P3 and miR‐206 inhibitor. Data were expressed as mean ± SD. *P < .05, **P < .01 represent statistically difference

Subsequently, bioinformatics tools showed that miR‐206 shared complementary binding sites with 3′‐UTR of ABCB1 mRNA (Figure 5A). Luciferase reporter assay showed that miR‐206 targeted with 3′‐UTR of ABCB1 mRNA with molecular binding (Figure 5B). In MCF‐7/PTX cells transfected with miR‐206 inhibitor, expression of both ABCB1 and FTH1P3 was up‐regulated (Figure 5C). Western blots showed that FTH1P3 silencing decreased ABCB1 protein expression, while FTH1P3‐enhanced expression and miR‐206 inhibitor promoted ABCB1 protein expression (Figure 5D and E). Overall, results indicated that FTH1P3 positively regulated ABCB1 expression via sponging miR‐206, suggesting the FTH1P3/miR‐206/ABCB1 pathway.

## 4 DISCUSSION

Breast cancer is one of the most common gynaecological malignancies for women and causes significant morbidity and mortality worldwide.^2^, ^15^, ^16^ Clinically, chemotherapy is the most effective assistant therapeutic method for the treatment of breast cancer.^17^ However, the occurrence of chemoresistance causes the poor prognosis, high recurrence rate and low 5‐year survival rate.^18^ In the present study, we investigate the role of lncRNA FTH1P3 in breast cancer paclitaxel resistance and explore the underlying mechanism for the drug resistance generation.

Up to now, emerging published literatures have indicated the important function of long non‐coding RNAs (lncRNAs) in the chemoresistance of multiple tumours.^9^, ^19^, ^20^ For example, lncRNA RP11‐770J1.3 and TMEM25 were highly expressed in paclitaxel‐resistant human breast cancer cells (MCF‐7/PR), while their down‐regulated expression enhanced the sensitivity of MCF‐7/PR cells to paclitaxel and inhibited the expression of MRP, BCRP and MDR1/P‐gp.^21^ Our research team identifies the overexpression of lncRNA FTH1P3 in paclitaxel‐resistant breast cancer tissue and cell lines. FTH1P3 was up‐regulated in paclitaxel‐resistant samples compared to sensitive samples; besides, FTH1P3 expression is increased when treated with increasing concentration of paclitaxel. LncRNA FTH1P3 is an intronless pseudogene transcript transcribed from ferritin iron storage protein ferritin.^22^ FTH1P3 has been reported to regulate the tumorigenesis in other type of cancers. For example, FTH1P3 is overexpressed in oral squamous cell carcinoma (OSCC) and the ectopic expression of FTH1P3 facilitates cell proliferation and colony formation in OSCC cells by acting as a molecular sponge of miR‐224‐5p.^23^

Paclitaxel is one of the first‐line chemotherapy drugs for multiple human cancers, including breast cancer.^24^ Paclitaxel could induce the cell cycle arrest at G2/M phase and induce the cell apoptosis. To test the role of FTH1P3 on paclitaxel‐resistant breast cancer cells, gain‐ and loss‐of‐function assays were performed. Results indicated that FTH1P3 silenced expression attenuated the IC50 value of paclitaxel and induced the cycle arrest at G2/M phase. On the contrary, FTH1P3‐enhanced expression plays the opposite roles. Therefore, we could conclude that the abnormal overexpression of FTH1P3 participate in the chemotherapy resistance, promoting the paclitaxel resistance in breast cancer tumorigenesis. The most probable mechanism involved in the drug resistance might that FTH1P3 regulates the cell cycle control to compete with paclitaxel treatment. LncRNAs have been verified to modulate the multiple resistance generation, involving cell cycle modulation, multidrug‐resistant protein and so on.^25^ For instance, lncRNA XIST is overexpressed in human lung adenocarcinoma A549 cells and increases the chemosensitivity to cisplatin, proposing the function through the let‐7i/BAG‐1 axis for the responsible for cisplatin resistance.^26^

With the rapid development of bioinformatics tools and high‐throughput sequencing, more and more potential lncRNAs have been identified and the detailed mechanisms are disclosing. The most canonical theory for lncRNAs is the miRNAs “sponge,” performing as miRNAs absorbers to specifically attenuate the miRNAs abundance. Our team performed bioinformatics predictive tools to investigate the potential regulatory pathway of FTH1P3 in the paclitaxel‐resistant breast cancer cells. Results indicate that miR‐206 binds with the 3′‐UTR of FTH1P3, which is confirmed using luciferase reporter assay. Moreover, miR‐206 also targets with 3′‐UTR of ABCB1 mRNA. Because FTH1P3 and ABCB1 are both up‐regulated in paclitaxel‐resistant breast cancer cells, we could conclude that miR‐206 targets simultaneously with FTH1P3 and ABCB1. ABCB1 (P‐glycoprotein) is one of most canonical chemoresistance protein that involving multiple drug resistance in cancer.^27^, ^28^ Our results reveal that FTH1P3 might promote ABCB1 protein expression through sponging mmiR‐206 and subsequently activates the paclitaxel resistance in breast cancer.

In conclusion, above evidence reveals the vital role of lncRNA FTH1P3 in the paclitaxel resistance of breast cancer. FTH1P3 promotes the paclitaxel resistance in human breast cancer tumorigenesis by targeting miR‐206/ABCB1 axis, suggesting the novel molecular mechanism of breast cancer chemotherapy resistance.

## CONFLICTS OF INTEREST

All the authors declare that they have no competing interests.
